# *Pseudasphondylia tominagai*, a new gall midge species (Diptera: Cecidomyiidae) inducing flower bud galls on *Eleutherococcus spinosus* (Araliaceae) in Japan

**DOI:** 10.3897/BDJ.7.e35673

**Published:** 2019-07-09

**Authors:** Ayman Khamis Elsayed, Junichi Yukawa, Makoto Tokuda

**Affiliations:** 1 The Botanical Gardens, Graduate School of Science, The University of Tokyo, Tokyo, Japan The Botanical Gardens, Graduate School of Science, The University of Tokyo Tokyo Japan; 2 Laboratory of Systems Ecology, Faculty of Agriculture, Saga University, Saga, Japan Laboratory of Systems Ecology, Faculty of Agriculture, Saga University Saga Japan; 3 Department of Applied Entomology, Faculty of Agriculture, Alexandria University, Alexandria, Egypt Department of Applied Entomology, Faculty of Agriculture, Alexandria University Alexandria Egypt; 4 Entomological Laboratory, Faculty of Agriculture, Kyushu University, Fukuoka, Japan Entomological Laboratory, Faculty of Agriculture, Kyushu University Fukuoka Japan

**Keywords:** Asphondyliini, Asphondyliina, host-alternation, taxonomic key

## Abstract

**Background:**

The genus *Pseudasphondylia* (Diptera: Cecidomyiidae: Asphondyliini: Asphondyliina) comprises ten Palearctic, Oriental and Australian species associated with various hosts belonging to at least ten plant families.

**New information:**

A new species, *Pseudasphondylia tominagai* Elsayed & Tokuda n. sp., that induces flower bud galls on *Eleutherococcus spinosus* (L.f.) S.Y.Hu (Araliaceae) is described. This species is considered to alternate between host plants seasonally. A key to males of known *Pseudasphondylia* species is provided.

## Introduction

The genus *Pseudasphondylia* (Diptera: Cecidomyiidae: Asphondyliini: Asphondyliina), until now, comprised ten described species, eight of them in the eastern Palearctic, one in the Oriental and one in the Australian Region (Gagné and Jaschhof 2017). Unlike the monophagous genus, such as *Daphnephila* (Asphondyliina) (Tokuda et al. 2008), *Pseudasphondylia* has a wide host range across many different plant families, such as Actinidaceae, Apocynaceae, Caprifoliaceae, Cornaceae, Ebenaceae, Elaeocarpaceae, Hydrangeaceae, Lauraceae, Rutaceae and Sabiaceae (Gagné and Jaschhof 2017). Recently, we found an undescribed species on Araliaceae, yet another host family for *Pseudasphondylia*. This new species forms a flower bud gall on *Eleutherococcus spinosus* (L.f.) S.Y.Hu (Araliaceae) in Osaka and Mie Prefectures, central Honshu, Japan.

Amongst the ten previously described species, the life history has been surveyed for only five Japanese species. *Pseudasphondylia neolitseae* on *Neolitsea sericea* (Blume) Koidz. (Lauraceae), *P. elaeocarpi* on Elaeocarpus sylvestris var. ellipticus Hara (Elaeocarpaceae) and *P. rokuharensis* on *Viburnum dilalatum* Thunb. (Caprifoliaceae) are monophagous and univoltine (Yukawa 1974, Yukawa 1983, Takasu and Yukawa 1984, Yukawa and Masuda 1996, Tokuda and Yukawa 2005, Tokuda and Yukawa 2002, Yukawa et al. 1976). In contrast, *P. kiritanii* and *P. matatabi* are suspected to be bi- or multivoltine species, alternating between host plants seasonally, but summer to spring hosts for *P. kiritanii* and overwintering hosts for *P. matatabi* have not yet been discovered (Yukawa and Masuda 1996, Tokuda and Yukawa 2005).

The aim of the present study is to describe the new species of *Pseudasphondylia* found on *E. spinosus*. In addition, an identification key to males of *Pseudasphondylia* is provided and the possible life history pattern of the species is dicsussed.

## Materials and methods

### Collecting and Rearing Methods

**Figure 1.**
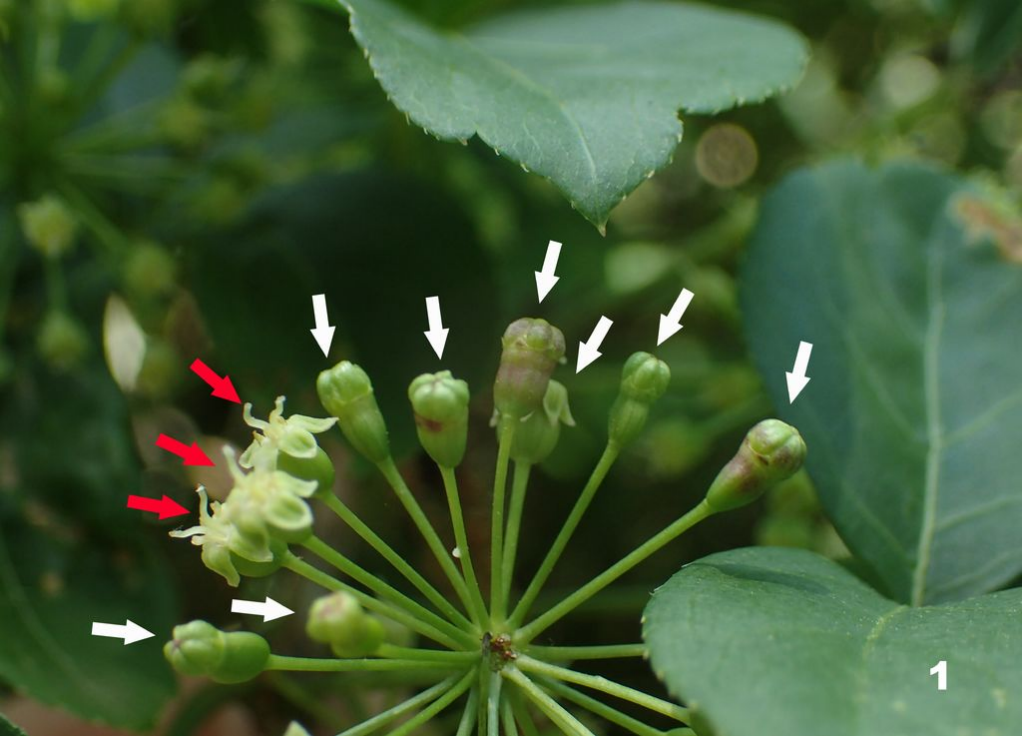
Flower bud galls (white arrows) of *Pseudasphondylia tominagai* n. sp. on *Eleutherococcus spinosus* (Araliaceae) [red arrows indicate normal flower buds].

Flower bud galls on *E. spinosus* (Fig. 1) were collected from two localities in central Honshu, Japan: Higashi-Osaka City, Osaka Prefecture and Misugi Town, Mie Prefecture. Some of the galls were dissected to obtain mature larvae, while others were kept in plastic bags until emergence of adults. Most of the specimens collected were preserved in 75% ethanol for morphological examinations.

### Morphological Examination and Terminology

Gall midge specimens were mounted on microscope slides in Canada balsam, following the technique outlined in Gagné (1994), except for the clearing step for some of the larval and adult specimens, following Elsayed et al. (2018). The slide–mounted specimens were examined under a bright–field and phase–contrast microscope (H550L, Nikon, Tokyo) and line illustrations were made with the aid of a drawing tube. Photomicrographs were taken with a digital camera (DP22, Olympus, Tokyo) attached to a semi-motorised fluorescence microscope (BX53, Olympus, Tokyo). Contrasts of some photographs and illustrations were adjusted and the figure plates were arranged using Adobe photoshop (version CS2, Adobe Systems).

Morphological terminology mainly follows McAlpine et al. (1981) for adults. The term “gonocoxal lobes” is used in accordance with Gagné (2018) and wing venation according to Yukawa (1971). Larval and pupal terminology follows Gagné (1994). All types of the newly described species are deposited in the collection of Entomological Laboratory, Faculty of Agriculture, Kyushu University, Japan (KUEC).

The new species was compared to specimens of five Japanese congeners in KUEC.

## Taxon treatments

### Pseudasphondylia tominagai

Elsayed and Tokuda, 2019 sp. n.

7b6d65ba-c796-57e6-8aff-d94898c3bdeb

urn:lsid:zoobank.org:act:3C010E4B-5CE3-4C2B-B280-21F4E70CF7D3

#### Description

Generic synopsis of *Pseudasphondylia* Monzen, 1955: see Tokuda and Yukawa (2005)

**Figure 2.**
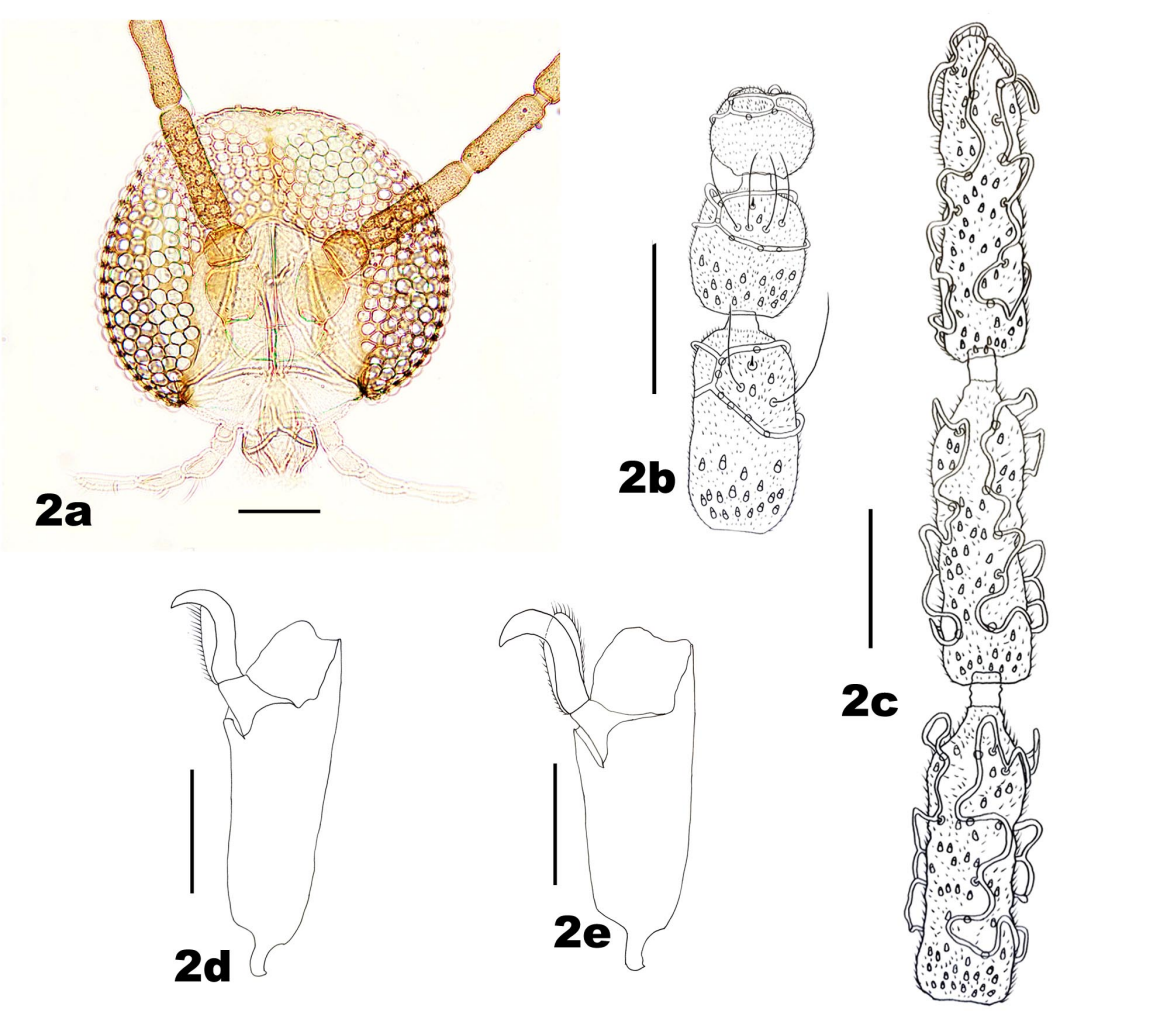
*Pseudasphondylia tominagai.* **a.** Head; **b.** Ventral view of female antennal flagellomeres X–XII; **c.** Ventral view of male flagellomeres X–XII; **d.** Tarsomere V and acromere of foreleg; **e.** Tarsomere V and acromere of hindleg. Scale bars = 50 µm.

*Adult*. Head (Fig. 2a–c) Eye bridge 6–8 facets long, facets rounded. Antenna: scape slightly wider than long, with scattered setae dorsally on the anterior two thirds and the posterior half ventrally; pedicel spheroid, with scattered setae on the anterior half; nodes of flagellomeres setose and microtrichose, with appressed circumfila and short, naked necks; female flagellomeres with two connected rings of circumfila, flagellomere I and II fused, flagellomeres I–IX elongate-cylindrical, becoming noticeably shorter successively, flagellomeres X–XII successively more foreshortened, flagellomere X twice as long as wide, flagellomere XI about 1.2 times as long as wide, flagellomere XII spheroid; male flagellomeres elongate-cylindrical, equal in length, with anastomosing wavy circumfila. Fronto-clypeus with 15–19 setae (n = 6). Palpus with noticeable palpiger, four-segmented, each segment with few setae and scales, first segment shortest (22–32 μm), second about twice as long as first, third about as long as second, fourth about 1.3 times longer than third. Labrum and labella setose and microtrichose.

Thorax: Anepisternum with 20–25 scales; anepimeron with 21–31 setae (n = 6); katepisternum bare. Lengths of leg parts as in Table 1; acropods (Fig. 2d–e): claws bent after midlength, less robust on foreleg than on mid- and hindlegs, empodia slightly shorter than claws, pulvilli diminutive. Wing (Fig. 3a): length 1.8–1.9 mm (n = 4) in male and 1.9–2.0 mm (n = 4) in female; width 0.88–0.92 mm (n = 4) in male and 0.92–1.04 mm (n = 4) in female; R_5_ joining C posterior to wing apex.

**Figure 4.**
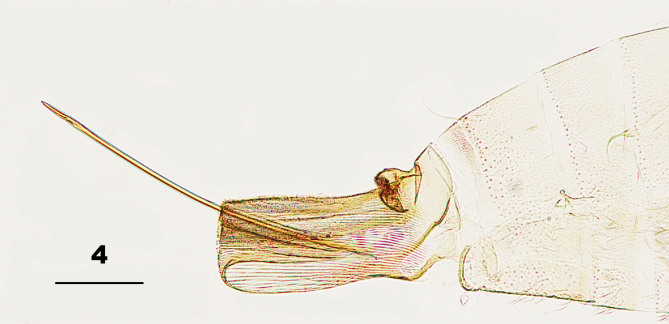
Terminal part of female abdomen of *Pseudasphondylia tominagai* n. sp. Scale bar = 50 µm.

Female abdomen (Fig. 4): Tergites I–VII rectangular, with anterior pair of trichoid sensilla and some lateral setae; tergite I with scales only on posterior half and posterior row of setae; tergites II–VII evenly covered with scales, tergites II–VI with single row of posterior setae; tergite VII with two posterior rows of setae; tergite VIII bare. Sternites II–VII with anterolateral pair of trichoid sensilla; sternites II–VI rectangular, with single row of posterior setae mixed with few scales, anterior two thirds with scattered setae and setiform scales; sternite VII about three times as long as VI, covered with scattered setae and scales. Ovipositor: eversible part with dorsal pair of pseudocerci basally; protrusible part needle-like, pigmented, about 2.7 (2.67–2.75; n = 4) times as long as sternite VII; cerci undifferentiated.

Male abdomen: Tergites I–VII and sternites II–VI as for female; tergite VIII band-like, bare, with no discernible trichoid sensilla; sternites VII–VIII with anterior pair of trichoid sensilla, covered with scattered setae and scales, sternite VII width about as for VI, sternite VIII about 0.7 as wide as VII. Terminalia (Fig. 5a): gonostylus with setae dorsally and ventrally on distal two thirds; cerci ovoid with setose margins; hypoproct shorter than cerci, bilobed, each lobe with one posterodorsal seta and one ventral seta; gonocoxal lobes about 0.4 times as long as the hypoproct; aedeagus longer than cerci, tapered.

**Figure 6.**
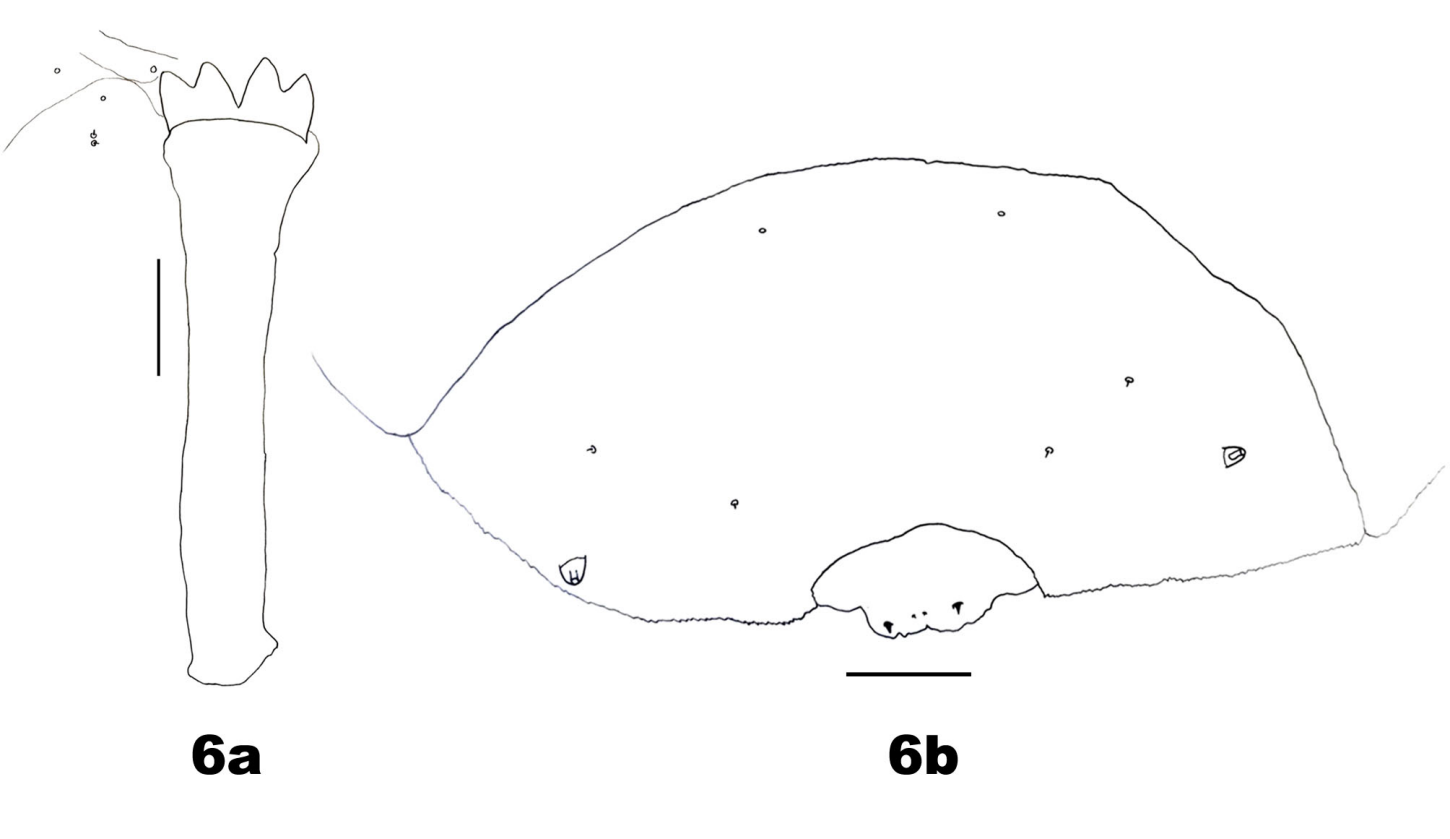
Larva of *Pseudasphondylia tominagai* n. sp. **a.** Spatula; **b.** Abdominal segment VIII and terminal segment dorsally. Scale bars = 50 µm.

*Third instar*. Pale yellow, body strongly bowed backwards. Spatula (Fig. 6a): quadridentate, inner teeth slightly longer than outer two; posterior portion about 3.7 times as long as width of anterior free portion. Three lateral papillae present, two with setae. Three asetose pleural papillae present anteriorly on each side of prothorax. Two pairs of asetose pleural papillae on meso- and metathorax. One pair of asetose pleural papillae on abdominal segments I–VIII. Two sternal papillae on each thoracic segment and abdominal segments I–VII, with setae, except on prothorax without setae. Two pairs of asetose elliptical papillae present anterodorsally on all thoracic and abdominal segments I–VIII. Two pairs of dorsal papillae present, without setae on thoracic segments and only outer pair with setae on abdominal segments I–VII. Terminal abdominal segment with two pairs of terminal papillae, outer pair with large setae and inner pair with minute setae (Fig. 6b). Four asetose anal papillae present.

**Figure 7.**
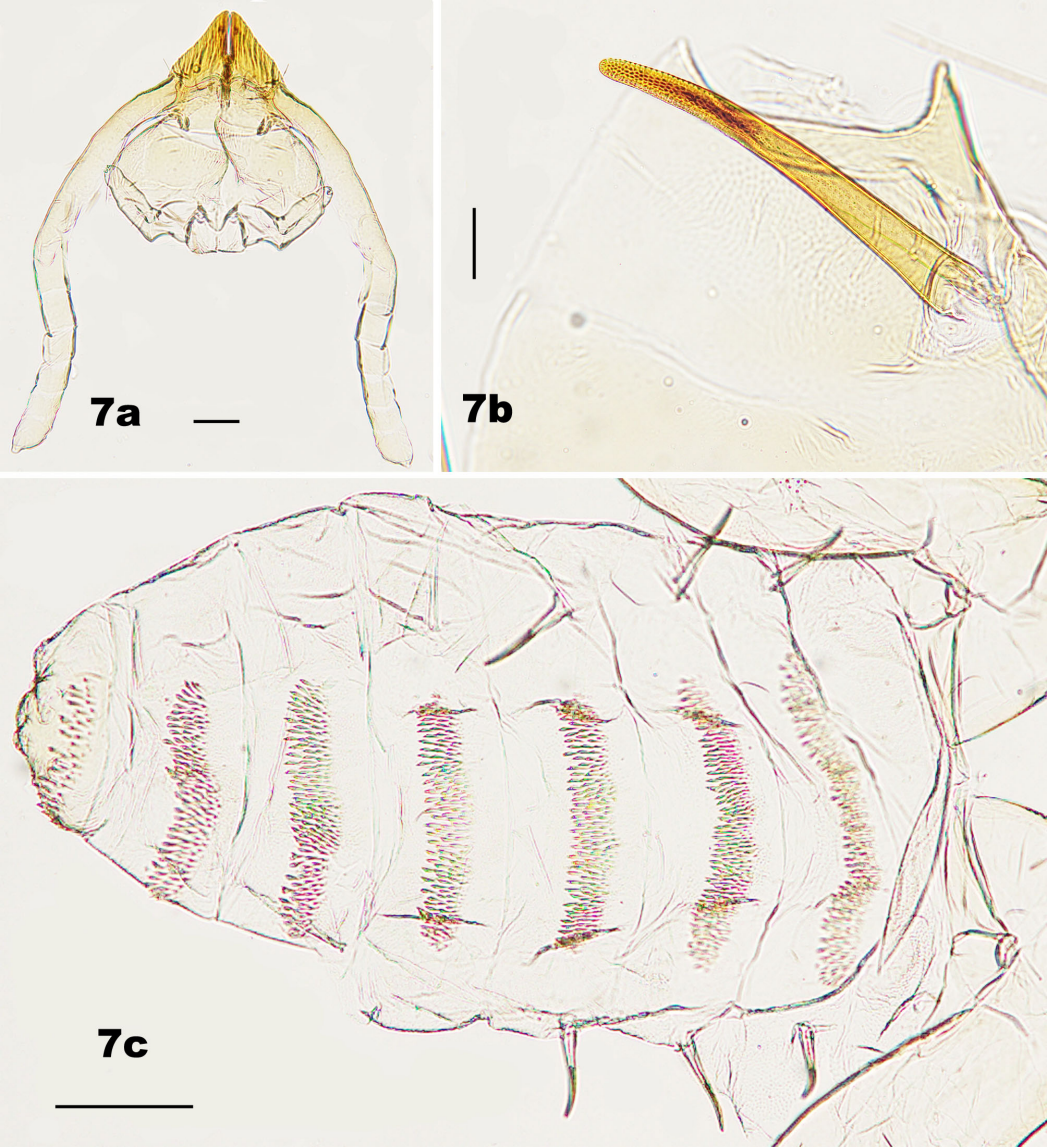
Pupa of *Pseudasphondylia tominagai* n. sp. **a.** Ventral view of head; **b.** Prothoracic spiracle; **c.** Dorsal view of abdomen. Scale bars = 50 µm.

*Pupa* (Fig. 7a–c). Four cephalic papillae present on tubercle, two with setae. Antennal horns greatly enlarged, tapered and flattened in ventral view, with serrate outer margins. One pair of setose lower facial papillae present. Two pairs of lateral facial papillae present, one pair with minute setae. Prothoracic spiracle elongated, slightly curved, about 320 μm long, with tracheae extending to tip. Abdominal spiracles present on segments II–IV, each spiracle about 0.5 times as long as the prothoracic spiracle. Abdominal terga I–VII with anterior pair of trichoid sensilla, 4–5 rows of spines and two pairs of dorsal papillae, only outer pair with setae; terga VIII with 3–4 rows of spines and two pairs of setose dorsal papillae.

**Etymology**: The species name, *tominagai*, honours Mr. A. Tominaga who collected the galls and reared the adults of this species.

**Holotype**: 1♂ (on slide): reared from flower bud gall on *E. spinosus*, collected in Misugi, Tsu City, Mie Prefecture, Japan, on 26.05.2018, emerged on 8.06.2018, A. Tominaga leg.

**Paratypes**: All paratypes (on slides) were reared from flower bud galls on *E. spinosus* in Japan by A. Tominaga. 4 larvae: galls collected in Misugi, Tsu City, Mie Prefecture on 26.05.2018, dissected on 26.05.2018; 4 larvae: galls collected in Higashi-Osaka City, Osaka Prefecture on 30.04.2018, dissected on 30.04.2018. 7 pupal exuviae, 5♀, 3♂: same data as holotype.

**Distribution**: Japan, Honshu: Osaka and Mie Prefectures.

**Gall and life history**: *Pseudasphondylia tominagai* induces flower bud galls on *E. spinosus*. The galled flower bud remains closed and reaches a diameter of 2.06–2.38 mm and length of 3.58–4.27 mm (n = 5) when matured. Larvae grow and pupate in the apical third of the galled bud. Third instars were found in the dissected galls in mid-April and adults emerged in late May.

#### Diagnosis

Amongst the five known *Pseudasphondylia* species in Japan, *P. tominagai* can be separated easily from *P. neolitseae*, *P. matatabi* and *P. elaeocarpi* at least by the following characters: narrower wings (Fig. 3d, e, f), more palpal segments and shape of sternal spatula (Yukawa 1971, Yukawa 1974, Tokuda and Yukawa 2005). It can be distinguished from *P. rokuharensis* as follows: wings of *P. tominagai* are slightly wider (Fig. 3c); the male of *P. tominagai* has the hypoproct with shallower-notch and the cerci more rounded and with deeper emargination in between; the female of *P. tominagai* has a longer ovipositor (the protrusible needle-like part of ovipositor is 2.7 times as long as sternite VII in *P. tominagai* compared to 1.8 times in *P. rokuharensis*) (Tokuda and Yukawa 2005); the pupa of *P. tominagai* has four dorsal papillae on abdominal segments I–VII, compared to six dorsal papillae in *P. rokuharensis* (Tokuda and Yukawa 2002); the larva of *P. tominagai* has four setose papillae on the terminal abdominal segment compared to only two in *P. rokuharensis*. *Pseudasphondylia tominagai* is closest to *P. kiritanii* because both species resemble each other in wing shape (Fig. 3a, b) and larval and pupal morphology; they can be distinguished from each other by the following characters: the male of *P. tominagai* has the hypoproct narrower than each cercus and with a shallower notch, whereas the hypoproct of *P. kiritanii* is slightly wider than each cercus and with a deeper notch (Fig. 5b); and the protrusible, needle-like part of the female ovipositor is slightly longer in *P. tominagai* (about 2.7 times as long as sternite VII) than in *P. kiritanii* (about 2.5 times as long as sternite VII).

## Identification Keys

### Key to males of known *Pseudasphondylia* species

**Table:** 

1	Palpus with fewer than four segments	2
–	Palpus with four segments	6
2	Palpus with three segments	3
–	Palpus with two segments	***P. neolitseae* Yukawa**
3	Empodia as long as tarsal claws	4
–	Empodia distinctly longer than tarsal claws (Mani 1954)	***P. campanulata* (Mani)**
4	Gonostylus with dorsal setae	5
–	Gonostylus without dorsal setae (Mo et al. 2007)	***P. zanthoxyli* Mo, Bu & Li**
5	Tergites I–VII with two rows of posterior setae	***P. matatabi* (Yuasa & Kumazawa)**
–	Tergites I–VII with a single row of posterior setae	***P. elaeocarpi* Tokuda & Yukawa**
6	Empodia as long as or slightly shorter than tarsal claws	7
–	Empodia distinctly longer than tarsal claws (Mo and Xu 1999)	***P. diospyri* Mo & Xu**
7	Cerci shallowly separated	8
–	Cerci deeply separated	9
8	Flagellomeres gradually becoming shorter from base to apex; hypoproct deeply notched (Coutin 1980)	***P. rauwolfiae* Coutin**
–	Flagellomeres equal in length; hypoproct shallowly notched (Kovalev 1964)	***P. philadelphi* (Kovalev)**
9	Each cercus wider than hypoproct, with rounded tips	10
–	Each cercus narrower than hypoproct, with pointed tips	***P. rokuharensis* Monzen**
10	Hypoproct deeply notched, slightly wider than each cercus	***P. kiritanii* Tokuda & Yukawa**
–	Hypoproct shallowly notched, narrower than each cercus	***P. tominagai* n. sp.**

## Discussion

In *Pseudasphondylia tominagai*, females of the overwintering generation lay eggs into flower buds of *E. spinosus*, which appear in March. Adult midges emerge from these flowers in late May when uninfested flower buds have already bloomed. Since we could not find any sign of larval presence in overwintering buds, we consider that *P. tominagai* possibly uses an alternative host plant from May to the following March.

In Diptera, the host-alternating habit has been known for only a few species of *Asphondylia*, such as *A. gennadii* (Marchal), *A. yushimai* Yukawa and Uechi, *A. baca* Monzen and *A. sphaera* Monzen (Harris 1975, Gagné and Orphanides 1992, Uechi et al. 2005, Uechi et al. 2004, Yukawa et al. 2003, Yukawa et al. 2016). Since *Asphondylia* and *Pseudasphondylia* belong to the same subtribe (Asphondyliina), they provide a great opportunity to study the evolutionary process of host-alternating habit by gall midges through molecular genetic analyses. Further taxonomic and ecological investigations are needed to clarify the life history of possible host alternating species in *Pseudasphondylia*.

## Supplementary Material

XML Treatment for Pseudasphondylia tominagai

## Figures and Tables

**Figure 3a.**
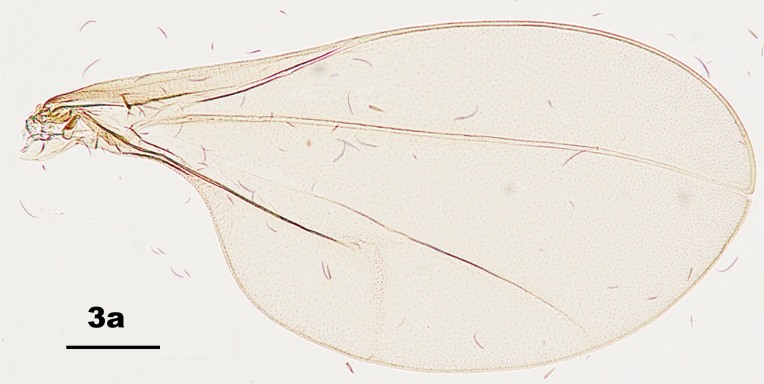
*P. tominagai* n. sp.

**Figure 3b.**
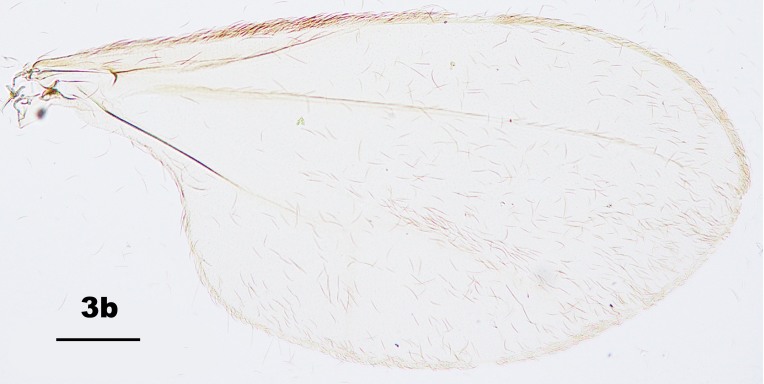
*P. kiritanii* Tokuda and Yukawa

**Figure 3c.**
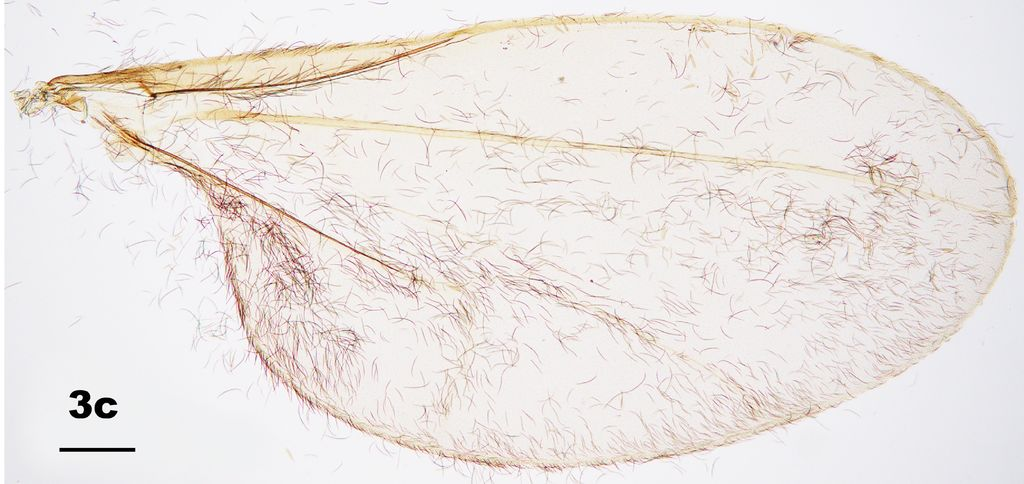
*P. rokuharensis* Monzen

**Figure 3d.**
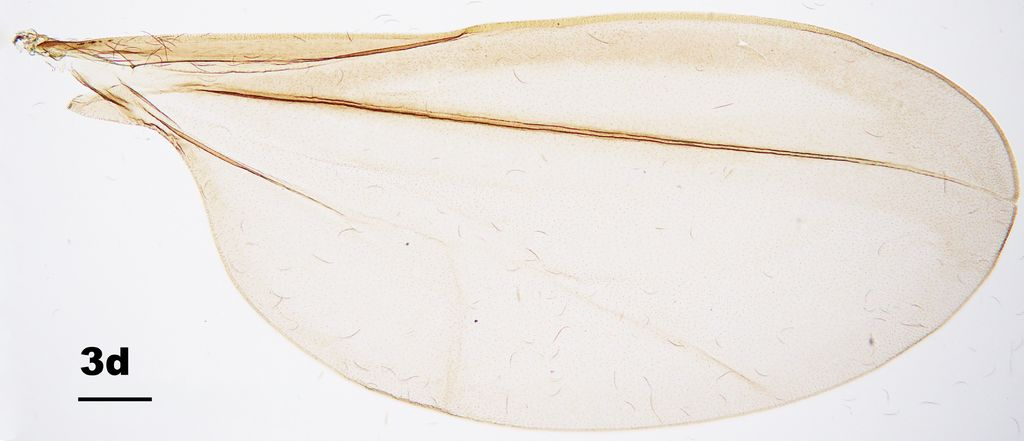
*P. neolitseae* Yukawa

**Figure 3e.**
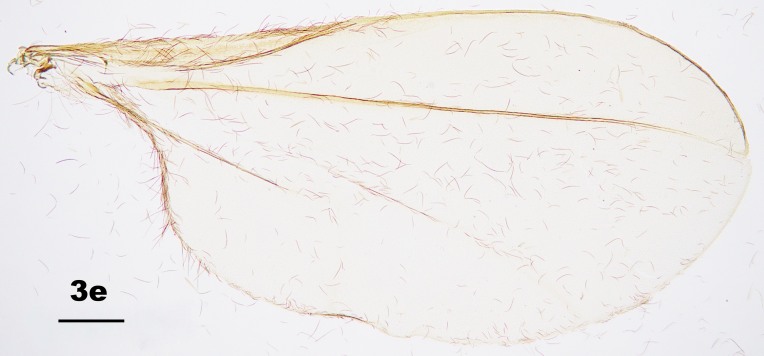
*P. matatabi* (Yuasa & Kumazawa)

**Figure 3f.**
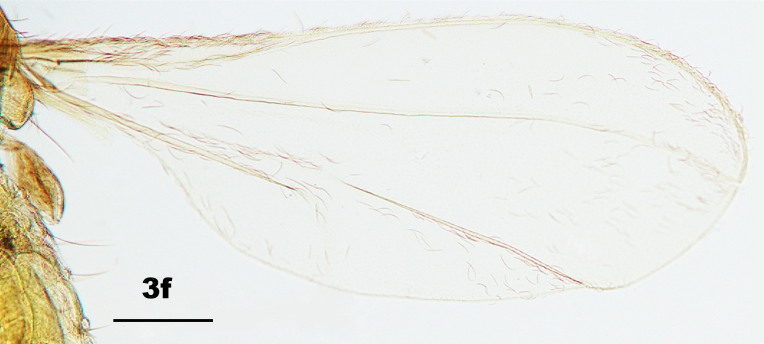
*P. elaeocarpi* Tokuda and Yukawa

**Figure 5a.**
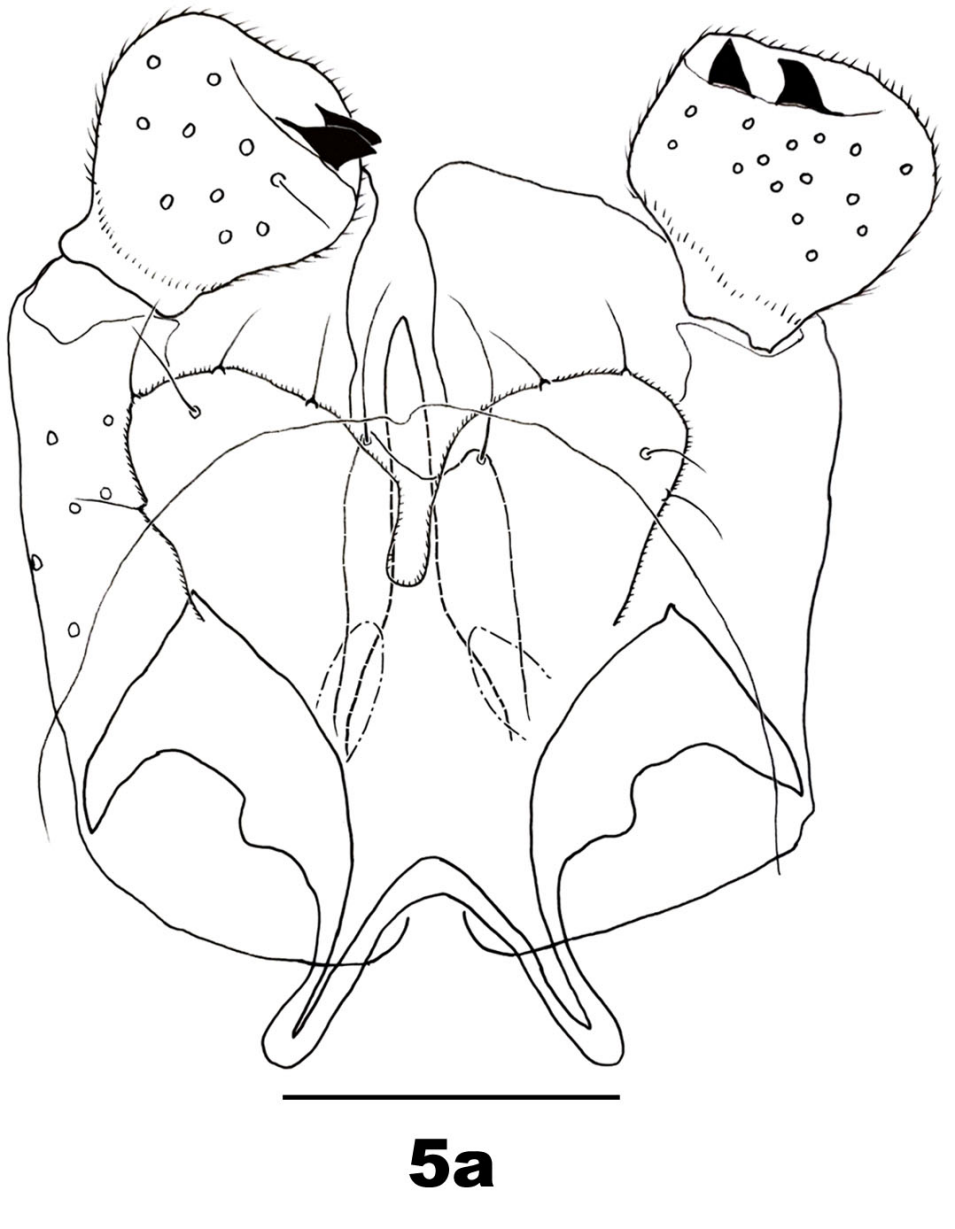
*P. tominagai* n. sp. (left gonostylus appears medially)

**Figure 5b.**
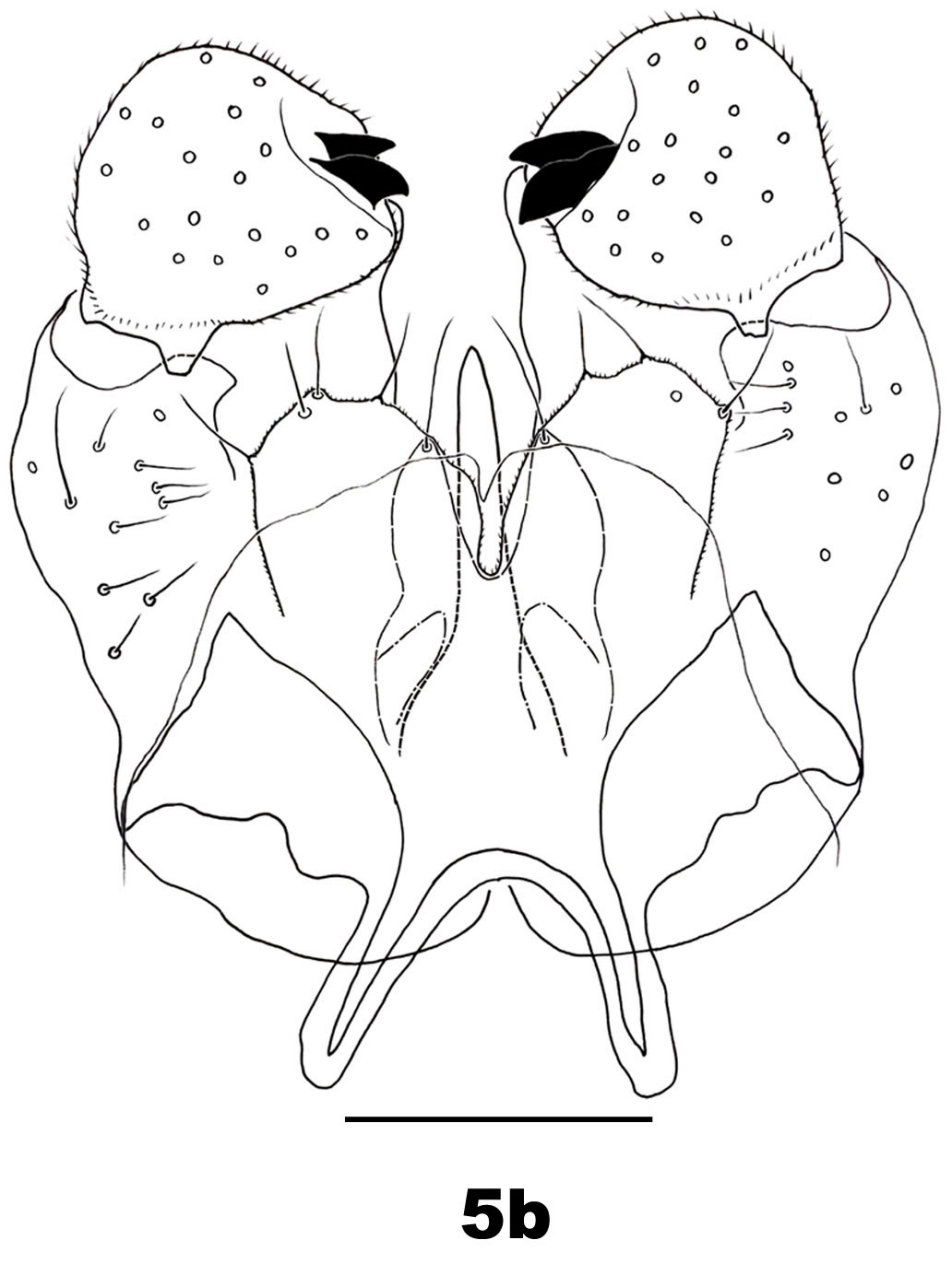
*P. kiritanii* Tokuda and Yukawa

**Table 1.** Leg length (µm) in *Pseudasphondylia tominagai*.

**Sex**		**Male (n=3)**		**Female (n=4)**	
		**Mean**	**Range**	**Mean**	**Range**
**Fore-leg**	**Fumer**	770	760-780	719	685-740
	**Tibia**	787	780-790	748	730-770
	**Tarsomere I**	117	110-120	122	120-125
	**Tarsomere II**	627	590-670	458	440-470
	**Tarsomere III**	307	300-310	194	180-210
	**Tarsomere IV**	203	190-210	125	115-140
	**Tarsomere V**	117	150-200	141	135-150
**Mid-leg**	**Fumer**	710	660-770	641	605-670
	**Tibia**	672	665-680	618	600-630
	**Tarsomere I**	107	105-110	120	120
	**Tarsomere II**	447	410-470	265	245-280
	**Tarsomere III**	253	250-260	143	135-150
	**Tarsomere IV**	160	150-170	96	90-100
	**Tarsomere V**	145	140-150	131	120-140
**Hind-leg**	**Fumer**	780	770-790	780	770-790
	**Tibia**	737	700-760	666	645-690
	**Tarsomere I**	110	110	124	120-130
	**Tarsomere II**	523	470-550	274	260-290
	**Tarsomere III**	310	300-320	149	140-160
	**Tarsomere IV**	197	190-210	106	100-110
	**Tarsomere V**	167	140-200	136	130-140
